# Gut microbiota related steroid hormone biosynthesis provide novel insights into high-salt diet related renal injury vit gut-kidney axis

**DOI:** 10.1186/s12866-025-04389-3

**Published:** 2025-10-21

**Authors:** Tian-hao Liu, Ting Xie, Yu-sheng Yu, Tong-tong Wang, Zhen-yu Bai, Sheng-yi Zhu, Yu-hao Niu, Li-guo Chen, Ya Xiao, Hong Wei, Chen-yang Zhang

**Affiliations:** 1 Department of Gastroenterology, Affiliated Hospital of Jiangnan University, Wuxi, Jiangsu China; 2 Department of Pathology, Army Medical University, Chongqing, China; 3 College of Chinese medicine, Jinan University, Guangzhou, Guangdong China; 4 Wuxi Medical College, Jiangnan University, Wuxi, Jiangsu China; 5 Yu-Yue Pathology Scientific Research Center, Jinfeng Laboratory, Chongqing, China; 6 The First Affiliated Hospital of Sun Yat sen University, Guangzhou, Guangdong China; 7 Institute of Integrated traditional Chinese and Western Medicine, Affiliated Hospital of Jiangnan University, Wuxi, Jiangsu China

**Keywords:** Gut-kidney axis, Gut microbiota, Renal injury, Steroid hormone biosynthesis pathway, Germ-free mice

## Abstract

High-salt diet (HSD) is a major risk factor for renal injury, and gut microbiota may play a role in this process. In this study, we investigated the potential role of gut microbiota in HSD-related renal injury and the microbial mechanisms involved. Through function observation, mechanism screening, and further verification using transcriptomic and metabolomic profiling and bioinformatics, we found that HSD caused renal dysfunction, inflammation, hypoimmunity, and serious renal damage in conventional mice, but this effect was absent in germ-free (GF) mice. Differential gene set enrichment analyses of the gut and kidney identified the steroid hormone biosynthesis pathway as a main culprit. For further verification, differential metabolite set enrichment analyses of feces indicated the involvement of the steroid hormone biosynthesis pathway. Through comprehensive profiling of intestinal and renal tissues along with fecal samples, we detected three genes and two metabolites showing prominent enrichment in the steroid hormone biosynthesis pathway. RT-qPCR suggested that the core gene *Cyp1a1*, which depends on the interplay between HSD and gut microbiota, was inhibited in both the gut and kidney in HSD-related renal injury. Finally, dehydroepiandrosterone decreased the mRNA expression of *Cyp1a1* in the gut and kidney. The data suggest that HSD promotes renal injury by manipulating the gut-kidney axis via gut microbiota and strengthening the steroid hormone biosynthesis pathway. The study expands the current knowledge on the gut microbial control of the gut-kidney axis in HSD-related renal injury, which finally provides novel insights into the therapeutic strategies for preventing or attenuating HSD-related kidney diseases.

**Supplementary Information:**

The online version contains supplementary material available at 10.1186/s12866-025-04389-3.

## Background

Chronic kidney disease (CKD) is a chronic disorder characterized by structural and functional kidney abnormalities caused by various factors. A decline in renal function is the most common symptom of CKD [1]. Approximately 10% of adults worldwide are affected by CKD, resulting in 12 million deaths and 28 million years of life lost yearly [2, 3]. CKD is expected to become the fifth leading cause of death worldwide by 2040, with one of the most significant anticipated rises of any major cause of death [4].

CKD has various origins, including those that are well-known and well-studied, such as diabetes, glomerulonephritis, and cystic kidney disorders, but the etiology of CKD is still unknown. The link between nutrition and chronic non-communicable diseases (NCDs) has received considerable attention. A global survey of all-cause mortality across 191 countries—including an analysis of 46 high-income nations—reveals that high-salt diet (HSD) is a significant risk factor for reduced life expectancy and shortened lifespan [5]. Furthermore, Franz H Messerli and others have demonstrated that salt intake in the diet is a risk factor for reducing life expectancy or causing premature death [5].

Long-term and excessive salt consumption harms organs and causes diseases by regulating blood pressure, boosting the immune system, and exacerbating inflammatory reactions, with the cardiovascular and kidney systems bearing the greatest impact [6]. Sodium consumption has been linked to high blood pressure and a higher risk of cardiovascular disease [7]. There is a link between gut microbiota and renal function, as well as HSD [8, 9]. Several studies have established a link between HSD and hypertension [8]. The ancient Chinese book *Synopsis of the Golden Chamber* records that salty taste corresponds to the health and disease of the kidney, and excessive salty taste will hurt the kidney.

The diversity, composition, and relative abundance of gut microbiota are all affected by HSD [10]. The relevance of the gut-kidney axis in renal disorders becomes most apparent [11, 12]. Therefore, this study aimed to explore the potential modulating role of gut microbiota on HSD-related renal injury and to elucidate the underlying microbial mechanisms based on transcriptomic, metabolomic profiling, and bioinformatics. The study provides novel insights into the therapeutic strategies for preventing or attenuating HSD-related kidney diseases.

## Materials and methods

### Experimental animals and groups

SPF C57BL/6J mice (male, 6–8 weeks old, provided by Beijing huafukang Biotechnology Co., Ltd.) and GF C57BL/6J mice (male, 6–8 weeks old, provided by the sterile animal research platform of the First Affiliated Hospital of Sun Yat-sen University) were divided into natural diet group (ND) and HSD group, with six mice in each group. Animal experiments were approved by the animal experiment ethics committee of the First Affiliated Hospital of Sun Yat-sen University and Jinan University (the ethics numbers: IACUC-2021503-1). All mice were housed at 20–24 ℃ and 50% relative Humidity with a 12-hour light/dark cycle.

### Experimental intervention

After ten days of quarantine and environmental adaptation, mice in the HSD group were given HSD feed (8% NaCl) for four weeks, whereas mice in the ND group were given control feed (0.4% NaCl) for the same duration [10, 13].

### Renal function-related factors

All procedures conformed to the guidelines from Directive 2010/63/EU of the European Parliament on the protection of animals used for scientific purposes. After four weeks of HSD intervention, the mice were anesthetized with isoflurane using a small animal ventilator (Shenzhen ruiward Life Technology Co., Ltd., No.: f8821-010) to avoid suffering. Blood was drawn from the orbital sinus and set in an aseptic blood collection vessel. Then the mice were euthanised using cervical dislocation under isoflurane anaesthesia. After blood was centrifuged for 5 min at 3000 rpm after 2–4 h, supernatants were collected in sterile 1.5-mL microcentrifuge tubes. The blood urea nitrogen (BUN) and serum creatinine (S-cr) levels were quantified using species-specific ELISA kits according to manufacturers’ protocols, with validation for non-serum samples. The renal tissue was then homogenized in 1% PMSF-NP40, centrifuged at 10,000 rpm at 4 ℃ for 15 min, and ELISA kits were used to detect tumor necrosis factor-α (TNFα), interleukin-6 (IL-6), interleukin-1β (IL-1β), interleukin-10 (IL-10), secretory Immunoglobulin A (SIgA), Na^+^/H^+^ exchanger type 3 (NHE3) in the supernatant of renal tissue. All ELISA kits were provided by MEIMIAN (Yancheng, China).

### Observation of pathological changes in intestinal tissues by using hematoxylin and eosin (HE) and masson staining

A mouse was randomly selected from each group, and renal tissues were collected to be conventionally immersed in 4% paraformaldehyde at room temperature for one night, routinely dehydrated, embedded, prepared, and stained with HE and Masson. The specimens were then observed under an optical microscope (microscope model: NIKON Eclipse ci; imaging system: NIKON digital sight DS-FI2). Each slice in each group selected three fields of vision to take photos, and as many tissues as possible filled the entire field of vision to ensure that the background Light of each photo was consistent with Image-Pro Plus 6.0.

### RNA-seq sequencing

#### RNA extraction

We extracted total RNA from renal and colonic tissues using TRIzol^®^ Reagent according to the manufacturer’s instructions (Invitrogen, USA), and genomic DNA was removed using DNase I (Takara). The RNA’s quality was then determined with 2100 Bioanalyser (Agilent) and quantified with ND-2000 (NanoDrop Technologies). Only high-quality RNA samples (OD260/280 = 1.8 ~ 2.2, OD260/230 ≥ 2.0, RIN ≥ 6.5, 28 S:18 S ≥ 1.0, > 1 µg) were used to construct the sequencing library.

#### Library preparation and illumina Hiseq xten/nova seq 6000 sequencing

RNA-seq transcriptome library was prepared using a TruSeqTM RNA sample preparation Kit from Illumina (San Diego, USA) using 1 µg of total RNA. Following quantification by using TBS380, the paired-end RNA-seq sequencing library was sequenced with the Illumina HiSeq xten/NovaSeq 6000 sequencer (2 × 150 bp read length).

#### Differential expression analysis and functional enrichment

To detect DEGs (differentially expressed genes) between two groups, we utilized the transcripts per million reads (TPM) method to calculate the expression level of each transcript. Gene abundances were quantified using RSEM (http://deweylab.biostat.wisc.edu/rsem/). Functional enrichment analyses, including KEGG, were performed to determine which DEGs were significantly enriched in metabolic pathways. KEGG pathway analyses were conducted utilizing Goatools (https://github.com/tanghaibao/Goatools) and KOBAS (http://kobas.cbi.pku.edu.cn/home.do).

### UPLC-MS/MS analysis

#### Metabolite extraction and quality control sample

Intestinal content samples were accurately weighed, and the metabolites were extracted using a solution of 400 µL methanol: water (4:1, v/v). The mixture was allowed to settle at −20 ℃ and treated with a high throughput tissue crusher Wonbio-96 c (Shanghai wanbo biotechnology co., LTD) at 50 Hz for 6 min, followed by vortex for 30 s and ultrasound at 40 kHz for 30 min at 5 ℃. The samples were then placed at −20 ℃ for 30 min to precipitate proteins. After centrifugation at 13,000 × g at 4 ℃ for 15 min, the supernatant was carefully transferred into sample vials for LC-MS/MS analysis. As a part of the system conditioning and quality control process, a pooled quality control sample (QC) was prepared by mixing equal volumes of all samples. The QC samples were disposed of and tested the same way as the analytic samples. It helped to represent the entire sample set, which was injected at regular intervals to monitor the stability of the analysis.

The chromatographic separation of the metabolites was performed on an ExionLC^TM^AD system (AB Sciex) equipped with an ACQUITY UPLC. The mobile phases consisted of 0.1% formic acid in water containing formic acid (0.1%) (solvent A) and 0.1% formic acid in acetonitrile : isopropanol (1:1, v/v)(solvent B). The solvent gradient changed based on the following conditions: from 0 to 3 min, 95% (A): 5% (B) to 80% (A): 20% (B); from 3 to 9 min, 80% (A): 20% (B) to 5% (A): 95% (B); from 9 to 13 min, 5% (A): 95% (B) to 5% (A): 95% (B); from 13 to 13.1 min, 5% (A): 95% (B) to 95% (A): 5% (B), from 13.1 to 16 min, 95% (A): 5% (B) to 95% (A): 5% (B) for equilibrating the systems. The sample injection volume was 20 µL, and the flow rate was set to 0.4 mL/min. The column temperature was maintained at 40 ℃. During the analysis period, all of the samples were stored at 4 ℃. The UPLC system was coupled to a quadrupole-time-of-flight mass spectrometer (Triple TOF^TM^5600+, AB Sciex) equipped with an electrospray ionization (ESI) source operating in positive mode and negative mode. The optimal conditions were set as follows: source temperature, 500 ℃; curtain gas (CUR), 30 psi; both Ion Source GS1 and GS2, 50 psi; ion-spray voltage floating (ISVF), −4000 V in negative mode and 5000 V in positive mode, respectively; declustering potential, 80 V; collision energy (CE), 20–60 V rolling for MS/MS. Data acquisition was performed with the Data Dependent Acquisition (DDA) mode. The detection was conducted over a mass range of 50–1000 m/z.

#### Data preprocessing and annotation

Following UPLC-TOF/MS analyses, the raw data were imported into the Progenesis QI 2.3 (Nonlinear Dynamics, Waters) for peak detection and alignment. The preprocessing results generated a data matrix consisting of retention time (RT), mass-to-charge ratio (m/z), and peak intensity values. At least 80% of metabolic features detected in any set of samples were retained. After filtering, minimum metabolite values were imputed for specific samples in which the metabolite levels fell below the lower limit of quantitation, and each metabolic feature was normalized using the sum rule. The internal standard was used for data QC (reproducibility); the metabolic features for which the relative standard deviation (RSD) of QC > 30% were discarded. Following normalization procedures and imputation, statistical analysis was performed on log-transformed data to identify significant differences in metabolite levels between comparable groups. The mass spectra of these metabolic features were identified using accurate mass, MS/MS fragments spectra, and isotope ratio difference by searching in reliable biochemical databases such as the Human metabolome database (HMDB) (http://www.hmdb.ca/) and Metlin database (https://metlin.scripps.edu/).

#### Multivariate statistical analysis

A multivariate statistical analysis was performed using ropls (Version1.6.2, http://bioconductor.org/packages/release/bioc/html/ropls.html) R package from Bioconductor on Majorbio Cloud Platform (https://cloud.majorbio.com). Principle component analysis (PCA) based on an unsupervised method was used to obtain an overview of the metabolic data, general clustering, trends, or to visualize outliers. All metabolite variables were scaled to unit-variances before performing PCA. Orthogonal partial least squares discriminate analysis (OPLS-DA) was used for statistical analysis to determine global metabolic changes between comparable groups. All metabolite variables were scaled to Pareto Scaling before conducting OPLS-DA. The model validity was evaluated from model parameters R2 and Q2, which provide information regarding the interpretability and predictability, respectively, of the model and avoid the risk of over-fitting. Variable importance in the projection (VIP) was calculated in OPLS-DA model. *P*-values were estimated using the paired Student’s t-test on single-dimensional statistical analysis.

#### Differential metabolites analysis

Statistically significant groups were selected with a VIP value of more than 1 and a *p*-value of less than 0.05. The differential metabolites between the two groups were summarized and mapped into their biochemical pathways through metabolic enrichment and pathway analysis based on a database search (KEGG, http://www.genome.jp/kegg/). The metabolites can be categorized based on the pathways or functions in which they participate. Enrichment analysis examines the presence or absence of a group of metabolites in a function node. The principle is that the annotation analysis of a single metabolite develops into an annotation analysis of a group of metabolites. scipy.stats (Python packages, https://docs.scipy.org/doc/scipy/) was used to identify a statistically significantly enriched pathway using Fisher’s exact test.

### Real-time quantitative PCR (RT-qPCR) assay

Total RNA from renal and colonic tissues was isolated using TRIzol^®^ reagent according to the manufacturer’s instructions (Invitrogen, USA), and genomic DNA was removed using DNase I (Invitrogen, USA). Each group was set with three repetitions. The quantity and purity of RNA were assessed through absorbance at 260 nm and 280 nm. Gene expression was measured in an ABI PRISM^®^ 7500 Sequence Detection System withSYBR Green qPCR SuperMix Kit (Promega). An endogenous control was set as GAPDH. The average Ct values from the triplicate analyses were normalized based on the average Ct values of GAPDH. The primer used in this study is detailed in Supplementary Table 1.

### Further animal validation experiments

Twelve SPF C57BL/6J mice (male, 6–8 weeks old), provided by Beijing huafukang Biotechnology Co., Ltd.), were divided into a control group and dehydroepiandrosterone group at the animal experiment center of Jiangnan University. Animal experiments were approved by the animal experiment ethics committee of Jiangnan University (the ethics numbers: JN.No20220930c0101224[378]). All mice were fed at 20–24 ℃ and 50% relative humidity, day and night. Dehydroepiandrosterone (Sigma-Aldrich) was dissolved in sterile corn oil at a concentration of 12 mg/mL for two weeks, once a day. Mice in the dehydroepiandrosterone group received intraperitoneal injections of this solution at 60 (dehydroepiandrosterone-low) or 120 (dehydroepiandrosterone-high) mg/kg/day. The control group received equal volumes of sterile corn oil alone. The drug dose and intervention time were set according to the results of the previous pilot study. The renal and intestinal tissues were used to perform HE, Masson, ELISA, and RT-PCR analyses. The detailed experimental methods were consistent with the analyses mentioned above.

### Statistical analyses

The data are presented as means ± standard error of the mean (SEM). A practical t-test method for comparison between two groups was conducted, and statistical charts were created using GraphPad Prism software. If *p*-value was less than 0.05, the difference was considered statistically significant.

## Results

### High dietary salt promotes renal injury development dependent on gut microbiota in mice

**Fig. 1:**
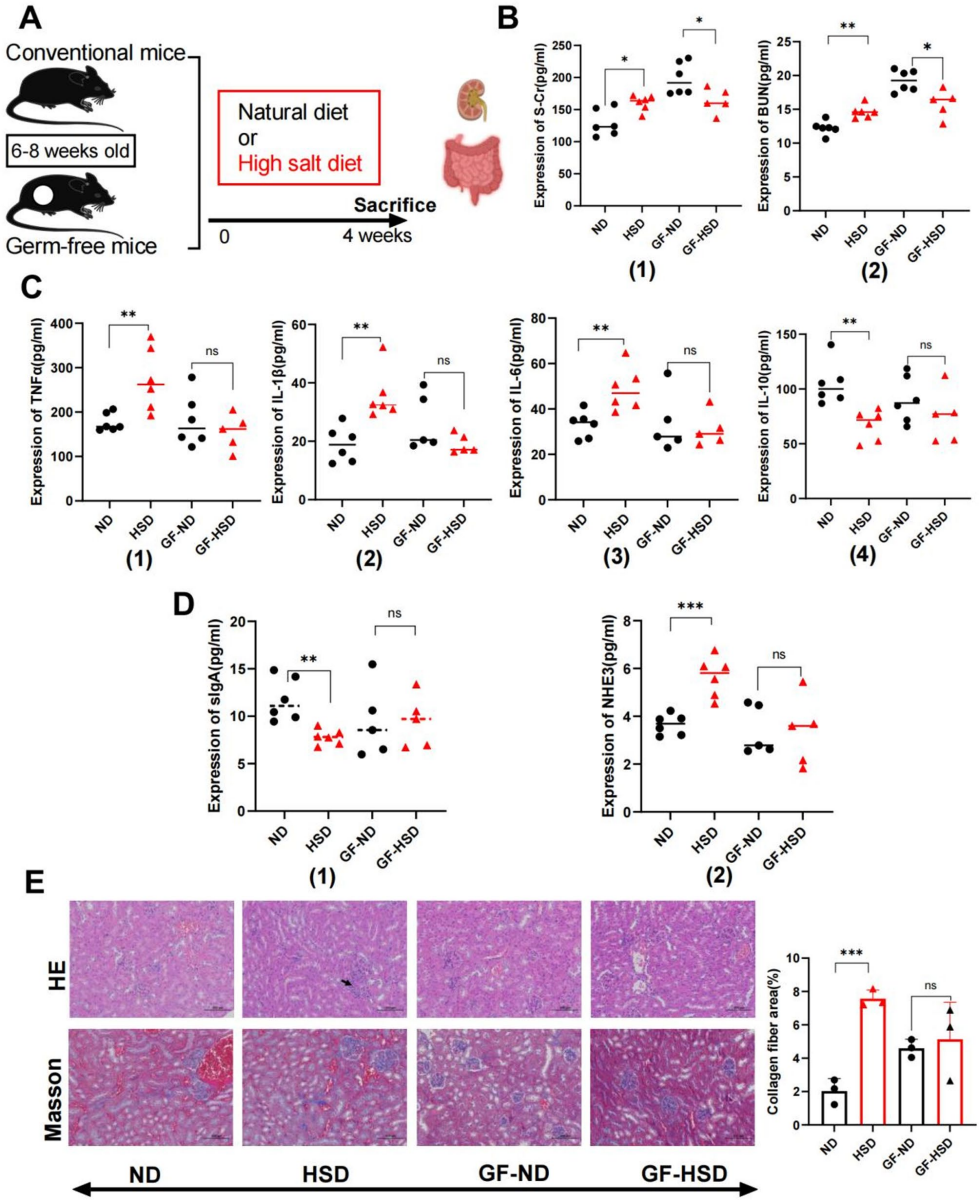
High dietary salt promotes renal injury development related to gut microbiota in mice.** A** Experimental design for dietary intervention. **B** Changes in renal function (S-Cr and BUN), measured using ELISA of mice fed the respective diets after four weeks (*n* = 5–6 mice per group, mean ± SEM; *p < 0.05, **p < 0.01, ***p < 0.001; ns, not statistically significant). **C** Changes in TNFα, IL-1β, IL-6 and IL-10, measured using ELISA of mice fed the respective diets after four weeks (*n* = 5–6 mice per group, mean ± SEM; *p < 0.05, **p < 0.01, ***p < 0.001; ns, not statistically significant). **D** Changes in sIgA and NHE3, measured using ELISA of mice fed the respective diets after four weeks (*n* = 5–6 mice per group, mean ± SEM; *p < 0.05, **p < 0.01, ***p < 0.001; ns, not statistically significant). **E** HE and Masson staining of kidney (scale bars, 20 μm), and quantitative assessment of collagen fiber (*n* = 3 per group, mean ± SEM; *p < 0.05, **p < 0.01, ***p < 0.001; ns, not statistically significant). ND: natural diet group; HSD: high salt diet group; GF: germ-free mice

To explore whether there are differences in the evaluation indexes of renal injury between conventional mice and GF mice under HSD conditions, the following experiments were carried out. The levels of renal function-related factors, including S-cr, BUN, TNFα, IL-6, IL-1β, IL-10, NHE3, and SIgA, in conventional and GF mice fed with HSD or natural diet for four weeks were measured (Fig. 1A). In conventional mice, HSD significantly increased the levels of S-cr and BUN in the kidney, on the contrary, the expression levels of S-cr and BUN significantly decreased in GF mice treated with HSD (Fig. 1B). These results indicated that the changes of inflammatory factors TNFα, IL-6, IL-1β and IL-10 in kidney tissue of mice. HSD resulted in significant increase in TNF-α, IL-6, and IL-1β, while a significant decrease (P value < 0.01) in IL-10 in the kidney tissue of conventional mice, but this was not the case for HSD-induced GF mice (Fig. 1C). SIgA was a mucosal immune antibody in the human body that contributes to the mucosal immunity of the intestinal tract against commensal bacteria [14]. NHE3 facilitated sodium absorption and proton secretion [15]. HSD significantly increased the level of NHE3 and decreased level of SIgA in the kidneys of conventional mice but not in GF mice (Fig. 1D). Hematoxylin-eosin staining was used to observe the pathological changes of the kidney (Fig. 1E). In conventional mice, HSD caused infiltration of inflammatory cells around the renal arterioles or renal interstitium, and eosinophils were observed in renal small cysts (Fig. 1E). However, no significant changes were found in GF mice (P value >0.05). The results of Masson staining indicated that HSD caused a significant increase (P value < 0.001) in collagen fibers in the renal tissue of conventional mice but insignificant changes in GF mice. The results of quantitative evaluation of collagen fibers (Fig. 1E) also showed that HSD caused a significant increase (P value < 0.001) in collagen fibers in the renal tissue of conventional mice, while the changes of collagen fibers in the renal tissue of GF mice were not obvious (P value > 0.05, Fig. 1E). These findings demonstrated that under the condition of HSD, the renal tissue injury of conventional mice was significantly serious, but the renal injury of GF mice was relatively mild.

### High dietary salt promotes the changes of gene expression profiling in the kidney dependent on gut microbiota in mice

**Fig. 2:**
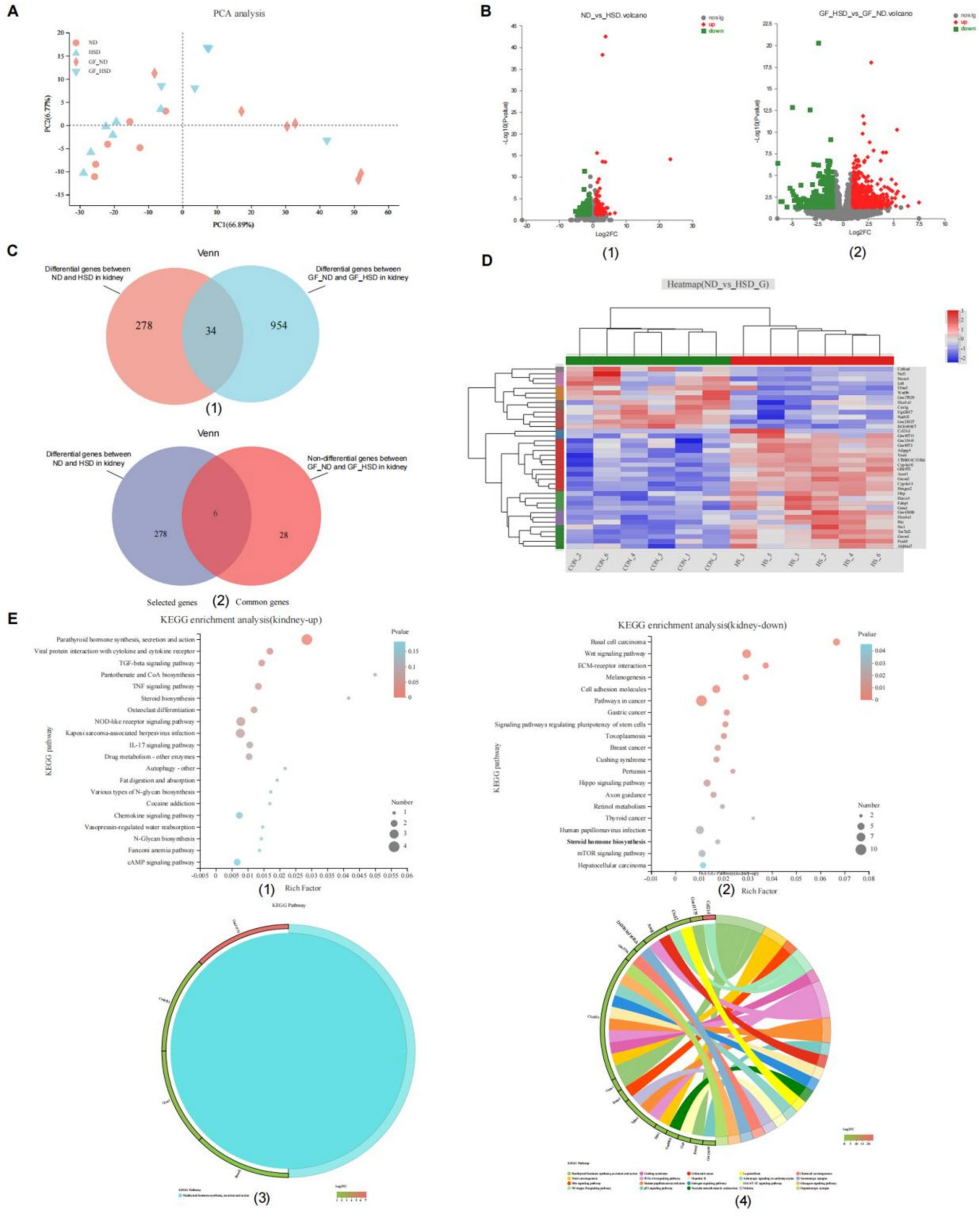
High dietary salt promotes the changes of gene expression profiling in the kidney related to gut microbiota in mice.** A** Principal-component analysis (PCA) plot explained 66.89% and 6.77% of the total variance with principle components (PCs) 1 and 2, respectively. Distances between plots reflect relative dissimilarities in community structures. **B** Differential gene expression in the kidney between ND versus HSD group. The up- or down-regulated gene was highlighted. **C **Venn diagram summarized gene changes in kidney samples in response to a high salt diet. **D** Heatmap analysis of the top 30 selected genes in the kidney between ND versus HSD group. **E** Histogram of KEGG classification. (1) Top 20 ranked pathways of KEGG enrichment based on the up-regulated genes. (2) Top 20 ranked pathways of KEGG enrichment based on down-regulated genes. (3) The significant differential of KEGG enrichment is based on up-regulated genes. (4) The significant differential pathways of KEGG enrichment are based on down-regulated genes

To investigate the differences in gene expression levels in renal tissues between conventional mice and GF mice under HSD conditions, and to identify the pathways involved in the unique up-regulated genes causing renal tissue damage in conventional mice, we performed the transcriptome sequencing of kidney tissue from the conventional and GF mice fed with HSD or ND. Principal-component analysis (PCA) plot reflected HSD change the kidney gene expression profiling in conventional and GF mice (Fig. 2A). The up- or down-regulated differential genes in the kidney were highlighted between ND and HSD groups (Fig. 2B). Among them, 312 differential genes (Supplementary Table 2, Standard: |log2fc| >1 and P value < 0.05) were identified between HSD and ND in conventional mice, of which 121 were up-regulated and 191 were down-regulated; while 988 differential genes (Supplementary Table 3, Standard: |log2fc| >1 and P value < 0.05) were identified between HSD and natural diet in GF group, with 325 up-regulated and 663 down-regulated (Fig. 2C; Supplementary Tables 2–3). Thirty-four common differential genes were screened in conventional and GF mice (Fig. 2C, Supplementary Tables 2–3). Considering that there may also be differential genes affected by HSD and gut microbiota in the intersection of the two differential gene sets, we constructed a differential gene set affected by high salt and gut microbiota, including 284 genes (Supplementary Table 4), of which 278 were differentially expressed only in conventional mice. (Fig. 2C, Supplementary Table 4). So we proposed a definition, and through the above experiments, it can be concluded that these 284 genes were unique genes closely related to kidney injury.

The gut microbiota is involved in the expression changes of certain genes in the kidney with HSD (Fig. 2D). The Kyoto Encyclopedia of Genes and Genomes (KEGG) database was used to differentially enrich upregulated and downregulated genes in the gene set to identify specific biological Functional pathways involved in differential genes related to interactions between gut microbiota and HSD. The 107 up-regulated differential genes were mainly enriched in parathyroid hormone synthesis, secretion and action, viral protein interaction with cytokine and cytokine receptor, and TGF-beta signaling pathway (Fig. 2E1). The chord diagram Further revealed that the detailed genes concluded in the 107 up-regulated differential genes were significantly enriched in the parathyroid hormone synthesis (Fig. 2E3). The 177 down-regulated differential genes were mainly enriched in basal cell carcinoma, the wnt signaling pathway, and ECM-receptor interaction (Fig. 2E2). The chord diagram Further proved that the detailed genes concluded in the 177 down-regulated differential genes were significantly enriched in the top 20 pathways, which included steroid hormone biosynthesis, a significantly different lipid metabolism-related pathway (Fig. 2E4). The results showed that there were differences in gene expression between conventional mice and GF mice under HSD conditions. The unique up-regulated genes of renal injury in conventional mice were distributed in the steroid hormone biosynthesis pathway.

### Changes in the gut gene expression profiling and functional analysis

Similarly, to investigate the differences in gene expression levels in intestinal tissues between conventional mice and GF mice under HSD conditions, and to identify the pathways involved in the unique up-regulated genes in intestinal tissue of conventional mice, we performed the transcriptome sequencing of intestinal tissues from the conventional and GF mice fed with HSD or natural diet.

**Fig. 3:**
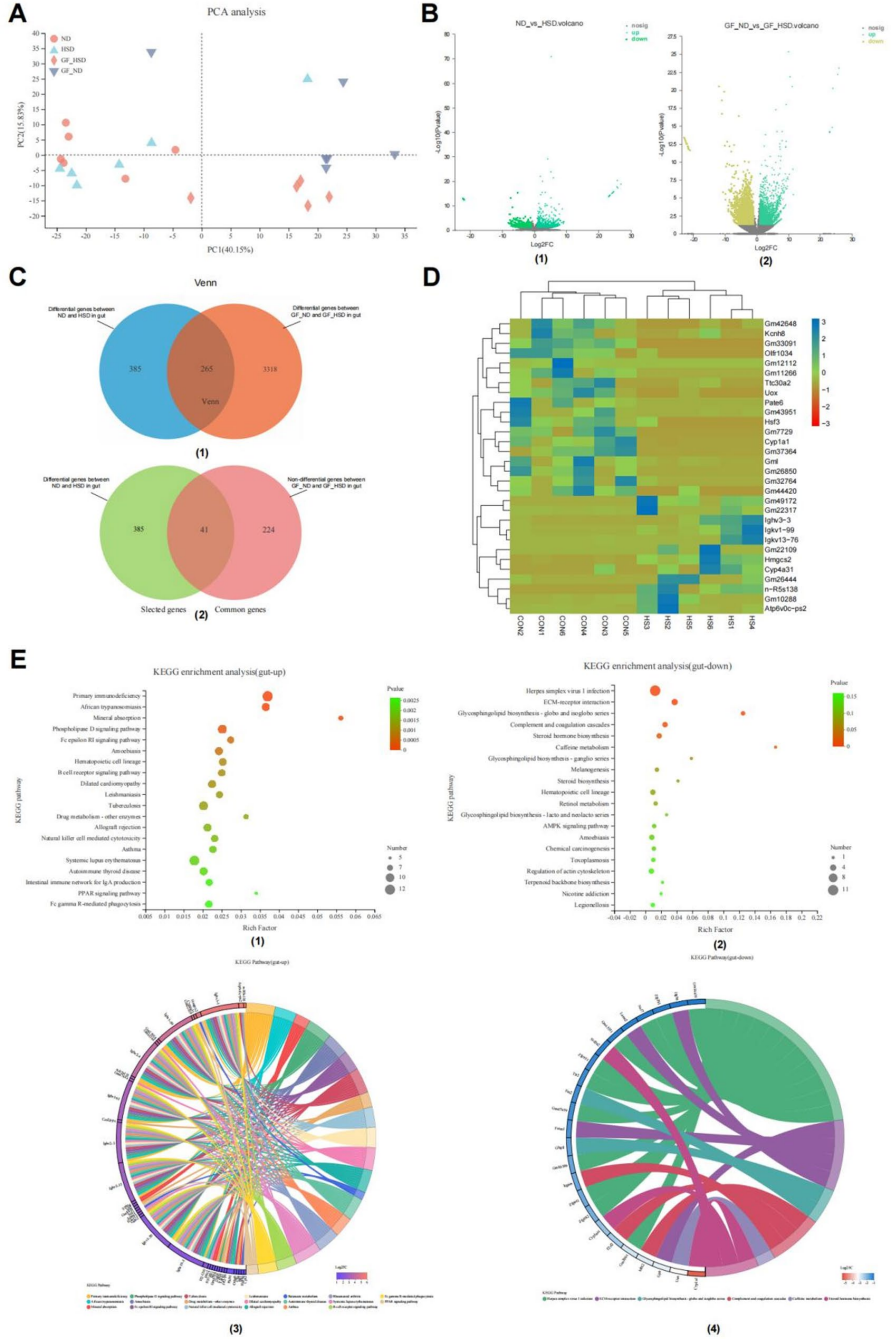
Gene expression profiling and functional analysis in the gut induced by high dietary salt related to gut microbiota in mice.** A** Principal-component analysis (PCA) plot explained 40.15% and 15.83% of the total variance with principle components (PCs) 1 and 2, respectively. Distances between plots reflect relative dissimilarities in community structures. **B** Differential gene expression in the gut between ND versus HSD group. The up- or down-regulated gene was highlighted. **C** Venn diagram summarized gene changes in gut samples in response to a high salt diet. **D** Heatmap analysis of the top 30 selected genes in the kidney between ND versus HSD group. **E** Histogram of KEGG classification. (1) Top 20 ranked pathways of KEGG enrichment based on up-regulated genes. (2) Top 20 ranked pathways of KEGG enrichment based on down-regulated genes. (3) The significant differential of KEGG enrichment is based on up-regulated genes. (4) The significant differential pathways of KEGG enrichment are based on down-regulated genes

Principal-component analysis (PCA) plot reflected HSD change the gut gene expression profiling in conventional and GF mice(Fig. 3A). The up- or down-regulated differential genes in the gut were highlighted between ND and HSD groups (Fig. 3B). Among them, 650 genes(Supplementary Tables 5–6, Standard: |log2fc| >1 and P value < 0.05) were identified between HSD and natural diet in conventional mice, of which 410 were up-regulated and 240 were down-regulated; while 3583 differential genes (Supplementary Tables 5–6, Standard: |log2fc| >1 and P value < 0.05) were identified between HSD and natural diet in GF group, of which 607 were up-regulated and 2976 were down-regulated (Fig. 3C; Supplementary Tables 5–6). The common 265 differential genes were screened in two states. Considering that there may be additional differential genes affected by HSD and gut microbiota in the intersection of the two differential gene sets, we constructed a differential gene set affected by HSD and gut microbiota, including 426 genes, of which 385 were differentially expressed only in conventional mice (Fig. 3C; Supplementary Table 7). The heatmap depicted the expression changes of the top differential genes from the differential gene library in conventional mice, further demonstrating that the gut microbiota is involved in the expression changes of certain genes in the kidneys with HSD (Fig. 3D). Differential enrichment of up-regulated and down-regulated genes in the gene set were performed using the KEGG database to reveal specific biological functional pathways involved in differential genes related to the interaction between gut microbiota and HSD (Fig. 3E). The 286 up-regulated differential genes were mainly enriched in primary immunodeficiency, African trypanosomiasis, and mineral absorption (Fig. 3E1). The chord diagram Further revealed the top 20 significant pathways enriched by the detailed genes concluded in the 286 up-regulated differential genes (Fig. 3E3). The 144 down-regulated differential genes were enriched mainly in Herpes simplex virus 1 infection, ECM-receptor interaction, glycosphingolipid biosynthesis—globo, and isoglobo series (Fig. 3E2). The chord diagram Further showed that the top 20 significant pathways enriched by the detailed genes concluded in the 286 up-regulated differential genes, including lipid metabolism-related pathway—steroid hormone biosynthesis (Fig. 3E4). Combined with the results in Fig. 2E4, the differential gene set up-regulated in intestinal and renal tissues, affected by the interaction between gut microbiota and HSD, were consistently significantly enriched in the steroid hormone biosynthesis pathway.The unique up-regulated genes that cause damage to the renal tissue of conventional mice share a common pathway with the distribution of unique up-regulated genes in the intestinal tissue of conventional mice, namely the steroid hormone biosynthesis pathway.

### Verification in the gut microbiota-related metabolic profiling and pathway-steroid hormone biosynthesis

To investigate whether there were differences in fecal metabolomics between conventional mice and GF mice under HSD conditions, and to identify unique differential metabolites in conventional mice under HSD conditions. We further observed the changes in metabolites of the colonic contents from the conventional and GF mice fed with HSD or natural diet through UPLC-TOF/MS analyses.

**Fig. 4:**
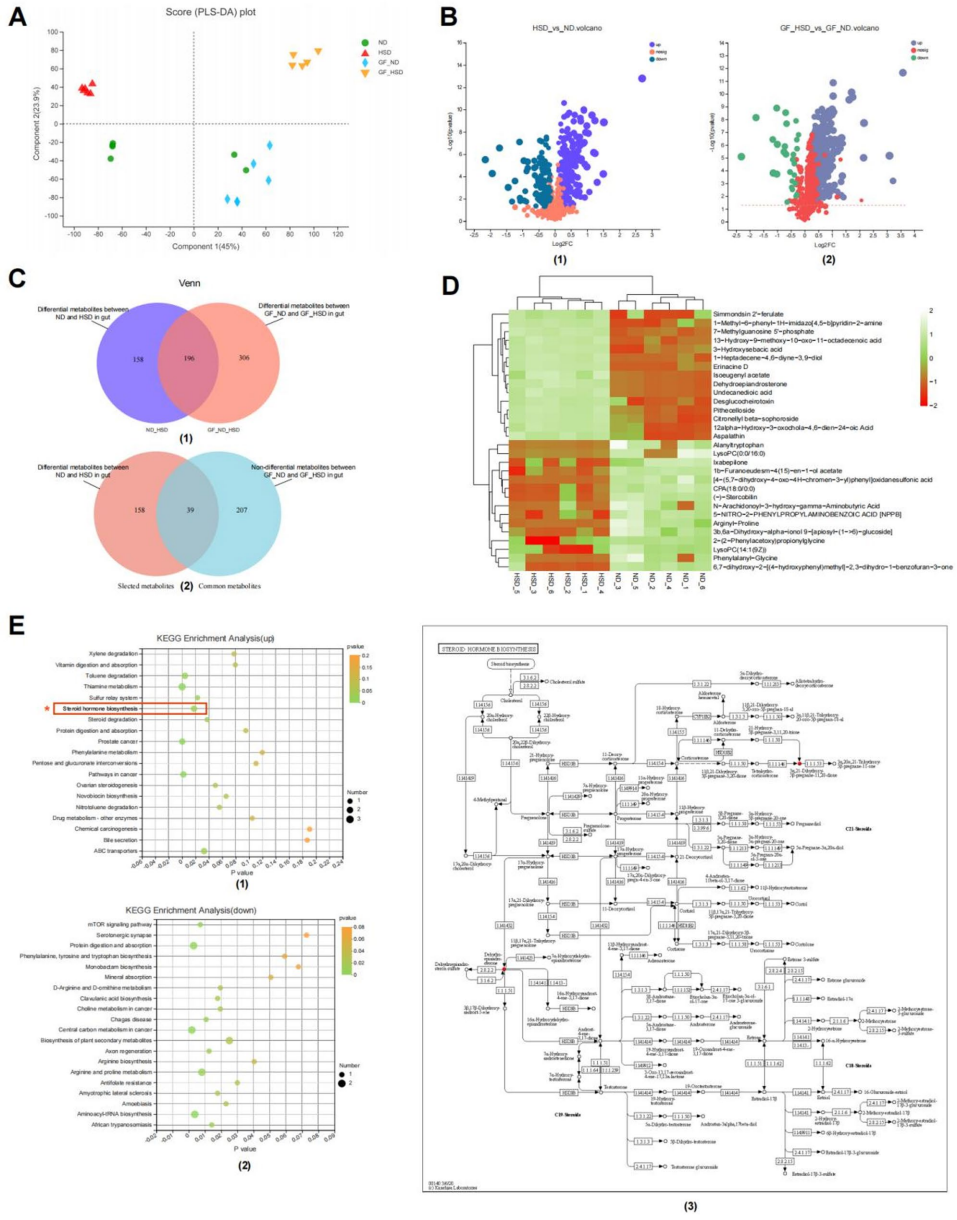
Metabolic profiling and pathway verified in the gut induced by high dietary salt related to gut microbiota in mice.** A** PLS-DA indicated 45% and 23.9% of the total variance with components 1 and 2, respectively. Distances between plots reflect relative dissimilarities in community structures. **B** Differential metabolite expression in the gut between ND versus HSD group. The up- or down-regulated metabolite was highlighted. **C** Venn diagram summarized metabolite changes in gut samples in response to high dietary diet. **D** Heatmap analysis of the top 30 selected metabolites in the gut between ND versus HSD group. **E** Histogram of KEGG classification. (1) Top 20 ranked pathways of KEGG enrichment based on up-regulated metabolites. (2) Top 20 ranked pathways of KEGG enrichment based on down-regulated metabolites. (3) The steroid hormone biosynthesis pathway was verified as a significant differential of KEGG enrichment based on up-regulated metabolites

PLS-DA indicated that HSD change the metabolic profiling in conventional and GF mice (Fig. 4A). The up- or down-regulated differential metabolite expression in the gut in conventional and GF mice fed with HSD or ND were highlighted (Fig. 4B). HSD led to significant differences in 354 metabolites (Supplementary Tables 8–9, Standard: |log2fc| >1 and *P* < 0.05) in conventional mice, of which 236 were up-regulated and 118 were down-regulated; HSD led to significant differences in 503 metabolites in GF mice, of which 475 were up-regulated and 28 were down-regulated (Fig. 4C; Supplementary Tables 8–9). The common 207 differential metabolites were screened in conventional and GF mice fed with HSD or ND. Considering that there may also be differential metabolites affected by HSD and gut microbiota in the intersection of the two differential metabolite sets, we constructed a differential metabolite pool affected by HSD and gut microbiota, including 197 metabolites, of which 158 were differentially expressed only in conventional mice (Fig. 4C; Supplementary Table 10). The heatmap depicted the expression changes of the top differential metabolites of the differential metabolite library in conventional mice, further demonstrating that the gut microbiota was involved in the changes in the expression of certain metabolites in the intestinal contents with HSD (Fig. 4D). To Further determine whether the differential metabolites could enrich gut microbiota-related pathway-steroid hormone biosynthesis, KEGG analyses based on 82 up-regulated and 115 down-regulated differential metabolites were performed (Fig. 4E). The results revealed that up-regulated differential metabolites might be significantly involved in thiamine metabolism, steroid hormone biosynthesis, and toluene degradation (Fig. 4E). The down-regulated differential metabolites may be significantly involved in the mTOR signaling pathway, central carbon metabolism in cancer, and arginine and proline metabolism (Fig. 4E). These findings confirmed that gut microbiota-related pathway-steroid hormone biosynthesis plays an essential role in HSD-related renal injury along with KEGG results in the gut and kidney. Under HSD conditions, there were differences in fecal metabolomics between conventional mice and GF mice, and differential metabolites closely related to kidney injury were found in conventional mice, which were involved in the steroid hormone biosynthesis pathway.

### Crosstalk between the screened gut microbiota-related metabolites and the host genes in steroid hormone biosynthesis pathway related to the gut-kidney axis.

**Fig. 5:**
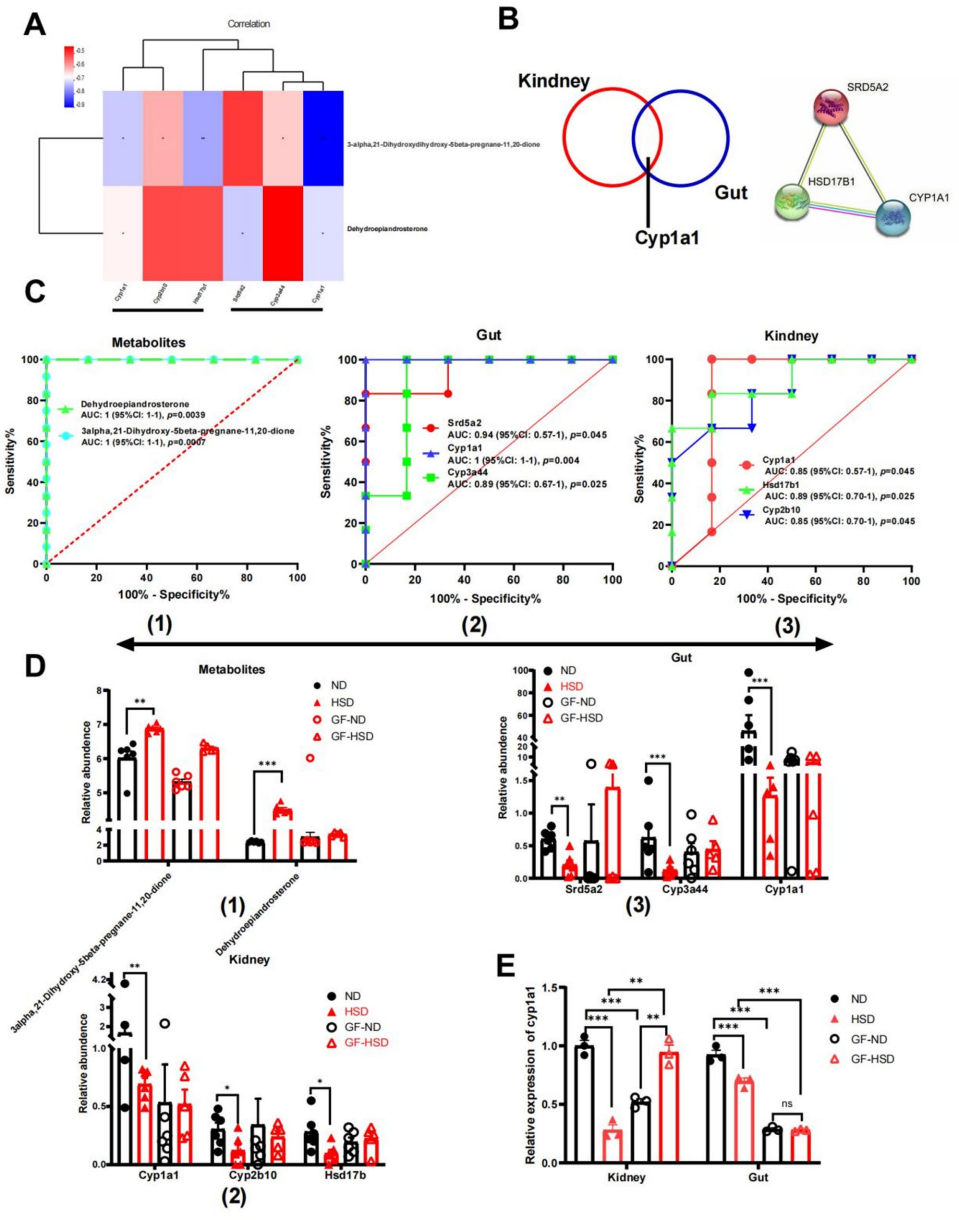
Crosstalk between the selected gut microbiota-related metabolites and the host genes related to gut microbiota in high dietary salt-induced mice.** A** The correlation analysis of selected differential metabolites and genes in the gut and kidney. The color represents the degree of correlation. **B** Protein-protein interaction (PPI) network of the total differential genes enriched in common steroid hormone biosynthesis pathway and venn diagram in gut and kidney. The annotation score demonstrated 0.955, 0.408, and 0.612 between CYP1A1 and HSD17B1, CYP1A1 and SRD5A2, HSD17B1 and SRD5A2, respectively. **C** The receiver operating characteristic curve (ROC) of selected differential metabolites and genes enriched in the common steroid hormone biosynthesis pathway. **D** The relative abundance of the selected differential metabolites and genes enriched in common steroid hormone biosynthesis pathway (*n* = 5–6 mice per group). **E** The *Cyp1a1* gene expression in intestinal and renal tissues using RT-qPCR (*n* = 3, mean ± SEM; *p < 0.05, **p < 0.01, ***p < 0.001; ns, not statistically significant). ND: natural diet group; HSD: high salt diet group; GF: germ-free mice

To elucidate the detailed correlation of the selected differential metabolites and genes in the gut and kidneys. The findings mentioned above revealed that the down-regulated differential genes in the kidney included *Cyp1a1*, *Cyp2b10*, and *Hsd17b1*, whereas the down-regulated differential genes in the gut included *Srd5a2*, *Cyp3a44*, and *Cyp1a1* (Supplementary Table 11). Furthermore, the above findings revealed that the up-regulated differential metabolites enriched in steroid hormone biosynthesis include 3-alpha,21-Dihydroxydihydroxy-5beta-pregnane-11,20-dione, and dehydroepiandrosterone (Supplementary Table 11). Therefore, we conducted the Spearman analysis between the differential genes and metabolites enriched in steroid hormone biosynthesis. The results clarified the detailed correlation of the selected differential metabolites and genes in the gut and kidney (Fig. 5A). Protein-protein interaction (PPI) network of the total differential genes enriched in common steroid hormone biosynthesis pathway in the gut and kidney, performed at STRING (https://cn.string-db.org) and is demonstrated in Fig. 5A. The results demonstrated 0.955, 0.408, and 0.612 in annotation scores between CYP1A1 and HSD17B1, CYP1A1 and SRD5A2, and HSD17B1 and SRD5A2, respectively (Fig. 5B). The results indicated that *Cyp1a1* might be the most important key gene among the five genes. The receiver operating characteristic curve (ROC) of the selected differential metabolites and genes enriched in the common steroid hormone biosynthesis pathway was performed based on HSD- or ND-induced conventional mice and is displayed in Fig. 5C (Standard: AUC:0.7–0.9, have a certain discriminatory effect; AUC > 0.9, have a good discriminative effect). Finally, the relative abundance of eight differential metabolites and genes related to steroid hormone biosynthesis pathway was performed in Fig. 5D. Increased relative abundance in 3-alpha,21-dihydroxy-5beta-pregnane-11,20-dione, and dehydroepiandrosterone was found in conventional mice fed by HSD compared with ND, whereas no changes were found in GF mice. The decreased relative expression of *Cyp1a1*, *Cyp2b10*, and *Hsd17b1* in the kidney and *Srd5a2*, *Cyp3a44*, and *Cyp1a1* in the gut were found in conventional mice fed by HSD compared with ND, whereas no changes were found in GF mice (Fig. 5C). We then selected the core genes of HSD and gutted microbiota involved in the gut-kidney axis for experimental verification using RT-qPCR to detect *Cyp1a1* gene expression in intestinal and renal tissues (Fig. 5E). HSD resulted in a significant decrease in *Cyp1a1* in renal and intestinal tissues of conventional mice, but this result was not observed in HSD-induced GFmice (Fig. 5E). It can be concluded that dehydroepiandrosterone can regulate *Cyp1a1* gene to induce renal injury. *Cyp1a1* is a key gene for gut microbiota to activate the gut-kidney axis in HSD-related renal injury. A further experiment verified that HSD induced low expression of *Cyp1a1* gene that plays an important role in the gut-kidney axis. These findings demonstrated that differential metabolites and genes enriched in the common steroid hormone biosynthesis pathway might act as a biomarker in HSD-related renal injury.

### Dehydroepiandrosterone produced by gut microbiota and high dietary salt inhibited the Cyp1a1 mRNA expression in the gut and kidney

**Fig. 6:**
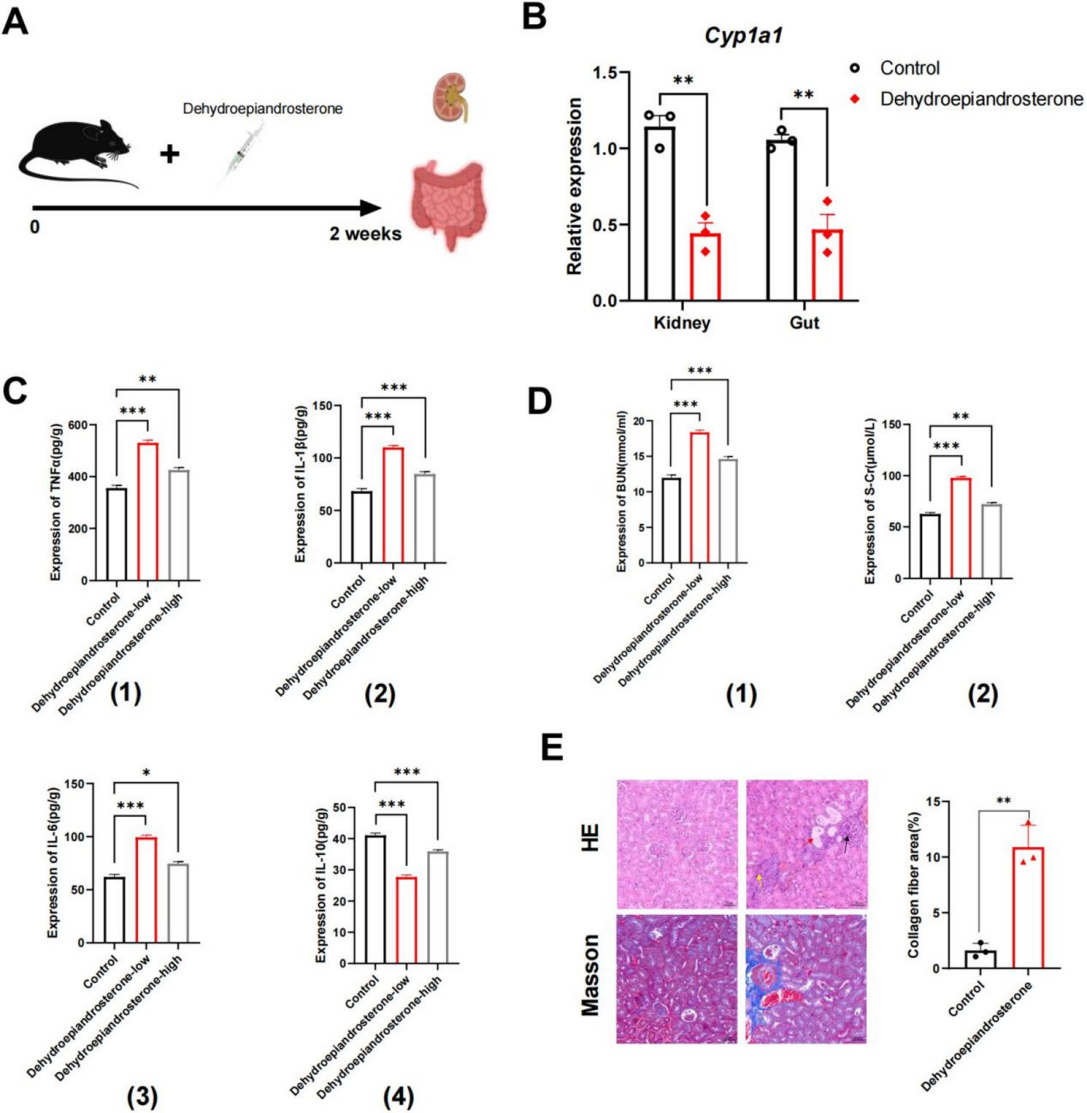
Dehydroepiandrosterone produced by gut microbiota and high dietary salt inhibited the ***cyp1a1*** expression in the gut and kidney.** A** Experimental design for dehydroepiandrosterone intervention. **B** *Cyp1a1* relative expression (*n* = 3, mean ± SEM; **p < 0.01). **C** Relative expression of TNFα, IL-1β, IL-6 and IL-10 (*n* = 8–10, mean ± SEM; *p < 0.05, **p < 0.01, ***p < 0.001; ns, not statistically significant). **D** Relative expression of S-Cr and BUN (*n* = 8–10, mean ± SEM; *p < 0.05, **p < 0.01, ***p < 0.001; ns, not statistically significant). **E** HE and Masson staining of kidney (scale bars, 20 μm), and quantitative assessment of collagen fiber (*n* = 3, mean ± SEM; **p < 0.01)

To further verify whether the enriched differential metabolite dehydroepiandrosterone and gene *Cyp1a1* in the steroid hormone biosynthesis pathway are biomarkers for HSD related kidney injury, additional animal experiments were conducted for dehydroepiandrosterone intervention, the levels of renal function-related factors, including S-cr, BUN, TNFα, IL-6, IL-1β, IL-10, in mice injected with dehydroepiandrosterone for two weeks were measured (Fig. 6A). Compared with the control group, a significant decrease (P value < 0.05) in *Cyp1a1* level in renal and intestinal tissues was found in mice injected with dehydroepiandrosterone for two weeks (Fig. 6B). Different doses of dehydroepiandrosterone resulted in a significant increase (P value < 0.0001) in the level of IL-6,TNF-α, IL-1β, S-cr, and BUN in renal tissue, but a significant decrease (P value < 0.0001) was observed in the levels of IL-10 (Figs. 6C-D). Hematoxylin-eosin staining (Fig. 6E) displayed that dehydroepiandrosterone led to abnormal renal tissue structure, including severe expansion of some renal tubules and filling with inflammatory cells (displayed by the red arrow), increased the number of glomerular mesangial cells (displayed by the black arrow), increased basophilia of renal tubular epithelial cells, and a small amount of inflammatory cell infiltration can be observed (depicted by the yellow arrow). The results of Masson staining demonstrated that dehydroepiandrosterone resulted in a significant increase in collagen fibers in the renal tissue (Fig. 6E). These findings demonstrated that dehydroepiandrosterone decreases the level of *Cyp1a1* in the gut and kidney and promotes renal injury development, verifying that core differential metabolites and genes enriched in the common steroid hormone biosynthesis pathway are related to HSD-related renal injury.

## Discussion

High salt intake is a risk factor for CKD and even hypertension [16]. The non-pharmacological strategy of diet modification to achieve renal protection is the cornerstone of CKD treatment [1]. The sulfur-containing amino acids in the diet are metabolized by gut microbiota to produce sulfide, which can be modified by sulfur-sulfhydrylation of tryptophan enzyme of *E. coli*, thereby reducing the generation of urinary toxin and renal injury in mice [17]. Through post-translational modification, diet can bring new enlightenment to tune microbiota function to support healthy host physiology [17, 18].

In this work, the interaction between HSD and gut microbiota was used to investigate the specific mechanism of gut microbiota activating the gut-kidney axis in HSD-related CKD. This work reveals that high dietary salt promotes renal injury development dependent on gut microbiota in mice. CKD typically appears alongside other comorbidities, highlighting underlying complex pathophysiology thought to be vastly modulated by the bidirectional gut-kidney crosstalk. CKD is a metabolic disease involving multiple tissue interactions, and its complex physiopathological and pathological functional changes are regulated by gut-kidney bidirectional regulation. The transplantation of fecal microbiota from mice on HSD into ordinary mice revealed that the gut microbiota in HSD group changed and induced renal injury and dysfunction, indicating that the interaction between diet and gut microbiota can be transmitted and changed and that the intestinal ecological imbalance induced by HSD is an important cause of renal injury [19]. Meanwhile, antibiotics can reverse the pathological features of intestinal leakage, early renal injury, and blood pressure rise induced by HSD in mice [19]. We discovered that HSD reduced the α diversity of the microbial community, reduced the relative abundances of *Rikenella* and *Christensenella*, and increased the relative abundances of *Atopobium* at the genus level in previous study [9]. Therefore, gut microbiota play the important role in HSD-related disease.

The renal injury involves pathophysiology, inflammation, immunity, and physiological functions. Most previous studies have revealed that HSD leads to the composition and structure of gut microbiota, immune dysfunction, and renal function injury [9]. Regulating macrophage function through the gut-kidney axis helps reduce renal fibrosis, which is a pathological change in chronic kidney disease [20]. Alterations in the gut-kidney axis have been found in CP-induced renal injury after enhancing the GLP-1 signaling pathway [21]. Manipulating the microbiota by oral administration of *oxalobacter formigenes* or probiotics containing inulin can prevent the formation of oxalate kidney stones, suggesting that manipulating the gut microbiota is a potential strategy for treating kidney stone diseases [22]. In our study, metabolomics, transcriptomics, and other methods were used to analyze the transcriptome characteristics of conventional and GF mice induced by HSD, the transcriptome characteristics of intestinal tissues, and the metabolic profiling of intestinal contents. Further, a combined analysis of transcriptomics and metabolomics was used to reveal the mechanism of gut microbiota in renal injury induced by HSD. First, this study revealed the changes in the renal transcriptome and intestinal transcriptome of gut microbiota involved in HSD-induced renal injury. Furthermore, the changes in metabolome were used to verify the molecular mechanism of gut microbiota regulating the gut-kidney axis involved in HSD-induced renal injury. Finally, the key genes and metabolites were verified in the present work.

The levels of Scr and BUN involved in evaluating the physiological function of the kidney, TNFα, IL-1β, IL-6, and IL-10, can be used to evaluate the inflammatory state of the kidney [23]. SIgA and NHE3 are related to the immune level and Na permeability [24, 25]. Our study discovered that HSD increased renal function-related factors, inflammatory response, and pathological damage in conventional mice. Compared with conventional mice, HSD did not cause pathological damage to renal function in GF mice, reflecting no changes in immunity, inflammation, or physiological function. Among them, elevated renal function factors were found in conventional mice, and decreased renal function factors were found in GF mice. These findings suggest that gut microbiota is a necessary intermediate medium for HSD-induced renal injury, and gut microbiota plays a promoting role. HSD led to the imbalance of gut microbiota and renal injury, and HSD cannot lead to renal injury without gut microbiota. Previous studies have found that antibiotics can reverse pathological features such as HSD-induced renal injury, consistent with our findings [19]. The loss or destruction of gut microbiota then blocked or reversed the protection of renal injury [19]. Furthermore, HSD has effectively inhibited tumor growth by up-regulating the number of NK cells and activation markers [26]. When we used antibiotics to destroy the gut microbiota of mice and fed them HSD, the results demonstrated that gut microbiota consumption induced by antibiotics destroyed the tumor inhibition and anti-tumor NK cell function mediated by HSD and that the gut microbiota was involved in regulating HSD and tumor [27].

To reveal the characteristics of renal transcriptome changes caused by gut microbiota participation in HSD, we first established the renal gene expression profiling changes of renal tissue involved in gut microbiota. For the first time, we systematically compared the changes of host transcription profiles in renal tissue of mice with or without gut microbiota interacting with HSD. We found 284 differential genes, including 107 up-regulated and 177 down-regulated, from the interaction between gut microbiota and HSD in the kidney. The up-regulated genes were significantly enriched in parathyroid hormone synthesis, secret, and action, and the down-regulated genes were significantly enriched in basal cell carcinoma, the wnt signaling pathway, ECM receiver interaction, and other top 20 signaling pathways. Parathyroid hormone is a key regulator of calcium and phosphorus homeostasis, which is related to CKD [28, 29]. Developing basal cell carcinoma is associated with constitutive activation of sonic hedgehog signaling, which is related to CKD, renal cell carcinoma, and clear cell renal cell carcinoma [30]. The wnt/β-catenin signaling pathway plays an important role in renal development and is re-expressed in the injured kidney [31]. ECM-receptor interactions associated with kidney development are also leveraged for regenerative bioactivity [32]. These findings indicate the potential mechanism in the renal injury induced by high dietary salt dependent on gut microbiota in mice.

To Further reveal the gut-kidney axis-related response mechanism of the interaction between gut microbiota and HSD, we established the gene expression profiling changes of intestinal tissue involved in gut microbiota and compared the changes of intestinal tissue transcription profiling of mice with or without gut microbiota and HSD for the first time. We found 426 genes in intestinal tissue, of which 286 were up-regulated and 140 were down-regulated. The up-regulated genes were significantly enriched in the pathways of primary immunodeficiency, African trypanosomias, mineral absorption, phospholipase D signaling pathway, etc. The down-regulated genes were significantly enriched in the pathways of Herpes simplex virus 1 infection, ECM receiver interaction, glycosphingolipid biosynthesis (globo and isoglobo series), and steroid hormone biosynthesis. In the axial relationship, a metabolic signal pathway can respond to two organs and tissues simultaneously. For instance, the MPAK signaling pathway reveals the axial relationship of the reno-cardiac axis [33]. Our study revealed that during the interaction between HSD and gut microbiota, steroid hormone biosynthesis responds synchronously in the down-regulated genes of the gut and kidney, which is also the only differential metabolic pathway with a common response, indicating that the steroid hormone biosynthesis pathway may be the key signal pathway of gut-kidney axis. No reports have confirmed the relationship between the steroid hormone biosynthesis pathway and the gut-kidney axis. Specifically, the steroid hormone biosynthesis pathway, the only common metabolism-associated pathway found in our work, belongs to the lipid metabolism pathways related to various diseases [34]. The adrenal cortex can synthesize various kinds of steroid hormones. The sex hormone produced by the adrenal gland is mainly dehydroepiandrosterone, which has a weak androgen effect and acts as other hormone precursors [35]. A combined transcriptome and metabolome analysis revealed that lovastatin, red yeast rice, and monascus pigment improved lipid metabolism in a high-fat model related to steroid hormone biosynthesis [36]. A previous study revealed that steroid hormone biosynthesis was significantly different between the benign tumor group and the renal cell carcinoma (RCC) group the urine metabolomes in a cohort of 61 patients with renal tumors and 68 healthy controls [37]. To verify whether the interaction between HSD and gut microbiota completes the gut and kidney interaction through the steroid hormone biosynthesis pathway, our work revealed 196 differential metabolites produced by the interaction between gut microbiota and HSD. The 88 up-regulated metabolites were enriched in thiamine metabolism, sulfur relay system, steroid hormone biosynthesis, protein digestion and absorption, and central carbon metabolism in cancer. The significant metabolites enrichment included the steroid hormone biosynthesis pathway. Therefore, to sum up, the interaction between HSD and gut microbiota completes the signal transmission of gut microbiota-related metabolites through the steroid hormone biosynthesis pathway, which provides strong scientific evidence to understand the significance of the gut-kidney axis in HSD-related CKD.

Furthermore, to identify the more core metabolites in the steroid hormone biosynthesis pathway, we first revealed the interaction between core genes through PPI network; second, we revealed the relationship between core metabolites and host genes using correlation analysis; third, these metabolites and genes can indeed be significantly distinguished using ROC curve; finally, the relative abundance of transcriptome and metabolome also further confirmed the differences of these metabolites (3-alpha,21-dihydroxy-5beta-pregnane-11,20-dione and dehydroepiandrosterone) and genes including *Cyp1a1*, *Cyp2b10*, *Hsd17b1*, *Srd5a2*, *Cyp3a44*. Currently, there is no report on the correlation between 3-alpha,21-dihydroxy-5beta-pregnane-11,20-dione and CKD. Dehydroepiandrosterone significantly reduced the basal expression of CYP1A1, and dehydroepiandrosterone inhibited the increase in hepatic CYP1A1 and CYP1A2 enzyme levels [38]. CYP1A1 is an important xenobiotic metabolizing enzyme associated with gene polymorphism in patients with CKD with unknown etiology [39]. To avoid differences caused by subjective factors, we selected the core genes of HSD and gut microbiota involved in the gut-kidney axis for experimental verification according to the following criteria: (1) they belonged to core genes in PPI network; (2) significant differences simultaneously existed between the two different metabolites; (3) AUC area in ROC analysis was larger; (4) renal and intestinal tissues responded simultaneously; (5) FC had a large variation multiple. Therefore, we used RT-qPCR to detect *Cyp1a1* gene expression in intestinal and renal tissues. A further experiment verified that HSD induced low expression of the *Cyp1a1* gene that plays an important role in the gut-kidney axis. In addition, we found that the sensitivity of *Cyp1a1* gene to HSD in GF mice is different from that in SPF mice, which may be related to the physiological functions of gut microbiota. Previous studies have shown that the gut microbiota influences CYP1A [40, 41]. The additional animal experiments demonstrated that dehydroepiandrosterone produced by gut microbiota and high dietary salt inhibited the *Cyp1a1* mRNA expression in the gut and kidney of mice, suggesting that the core differential metabolites and genes enriched in common steroid hormone biosynthesis pathway are related to HSD-related renal injury. Neuron-specific deletion of Ahr, or constitutive overexpression of its negative feedback regulator CYP1A1, results in a reduced peristaltic activity of the colon, similar to that observed in microbiota-depleted mice [42]. Actinomycin D chase experiments in T47D cells revealed that dehydroepiandrosterone increased the rate of CYP1A1 mRNA degradation [43]. Therefore, our present work suggests that the interaction mechanism of HSD and gut microbiota in HSD-related renal injury is closely associated with the steroid hormone biosynthesis pathway.

Our work provides more detailed evidence of the gut-kidney axis in HSD-related renal injury, the study confirms that HSD promotes renal injury by manipulating the gutkidney axis via gut microbiota and strengthening the steroid hormone biosynthesis pathway. Increasing evidence has demonstrated a bidirectional relationship between host and gut microbiota in patients with various kidney diseases^41^. Our findings provide new insights into therapeutic strategies to prevent or alleviate HSD-related kidney disease. Through in-depth study, we found that gut microbes play an important regulatory role in HSD-related renal injury. We found that by regulating the composition and function of the intestinal microbial community, we can effectively intervene in the development of HSD-related kidney disease. In addition, our study also revealed the interaction network in the gut-kidney axis, including the interaction between intestinal microbes and intestinal mucosal barrier and kidney. However, this study has several limitations: the use of only male mice creates a single-sex model; the HSD intervention followed a single fixed duration; and dynamic phenotype monitoring was lacking. Although elevated dehydroepiandrosterone was observed, the specific bacterial species responsible for its production and the regulatory mechanisms were not elucidated. These aspects warrant more comprehensive investigation in future research.

The findings provide important clues for us to further understand the regulatory mechanism of the gut-kidney axis. Based on these findings, we can explore new therapeutic strategies to prevent or alleviate the development of HSD-related kidney disease. It will help to reduce the pathological process of HSD-related kidney disease. Furthermore, we can also use the metabolites of intestinal microorganisms as potential therapeutic targets. By regulating the metabolic activity of the intestinal microbial community, it affects the signal transduction pathway in the gut-kidney axis, thereby improving the pathological process of HSD-related kidney disease.

Increasing evidence has demonstrated a bidirectional relationship between host and gut microbiota in patients with various kidney diseases [44]. Previous studies have demonstrated that the gut microbiota affects the expression of kidney genes in a gender-specific manner, which may affect the physiological function of the kidney, while, the related mechanism has not been confirmed. Even microbiota from patients transplanted to renal injured GF or antibiotic-treated rats induces higher production of serum uraemic toxins and aggravated renal fibrosis and oxidative stress more than microbiota from controls [45]. Therefore, the more detailed mechanisms were required further exploration.

## Conclusion

**Fig. 7:**
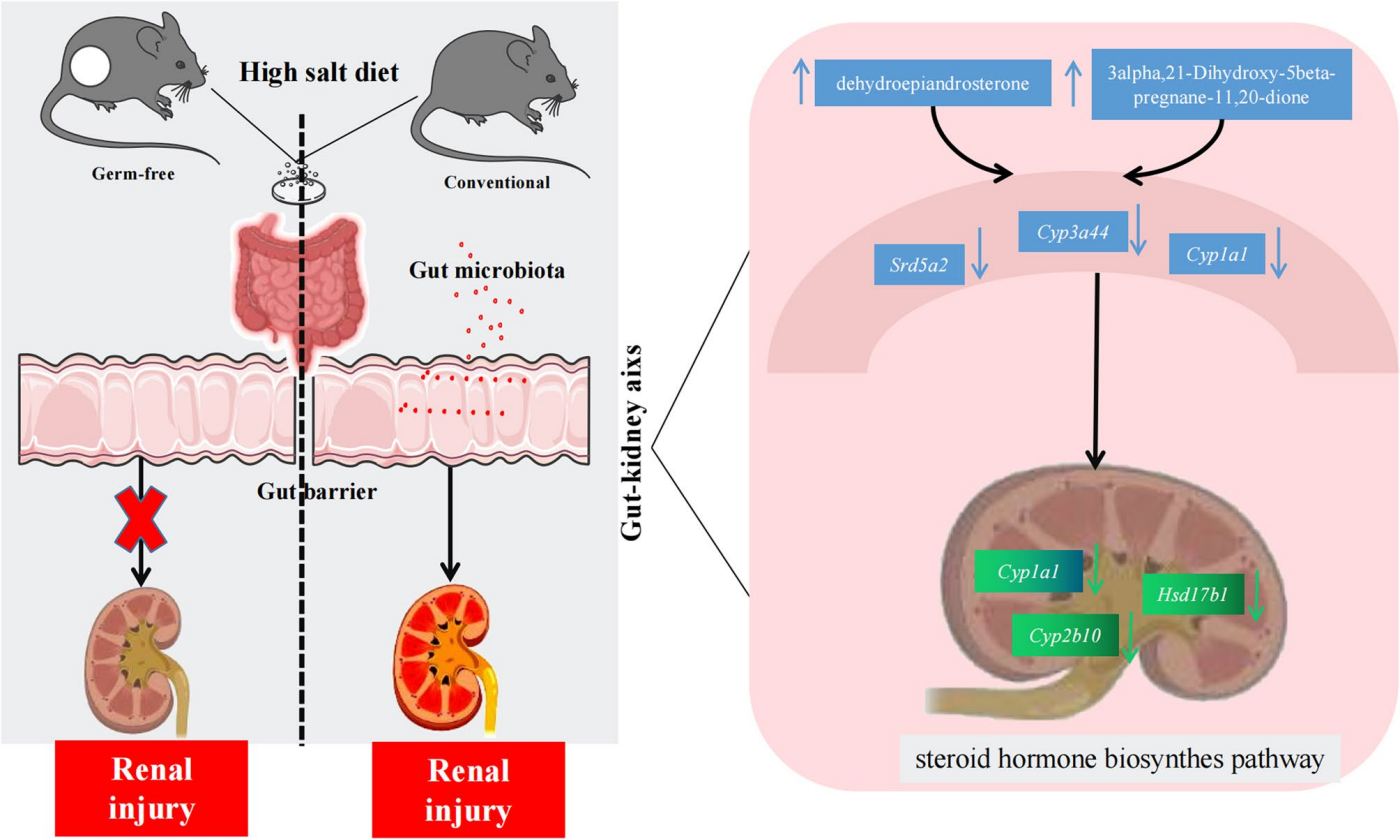
Schematic diagram representing the mechanism of gut microbiota and high dietary salt via modulating gut-kidney axis enriched in steroid hormone biosynthesis pathway

This study demonstrates that HSD can promote renal injury and necessarily depend on gut microbiota, with the research features of the GF mice model for the first time. Overall, our data suggest that the interaction mechanism of HSD and gut microbiota in HSD-related renal injury is closely associated with the steroid hormone biosynthesis pathway. In summary, this study highlights the potential role of gut microbiota in HSD-induced renal injury and identifies the steroid hormone biosynthesis pathway as a key mechanism involved (Fig. 7). These findings suggest that targeting the gut microbiota and steroid hormone biosynthesis pathway may be potential therapeutic strategies for preventing or treating HSD-related renal injury.

## Supplementary Information

Supplementary Material 1.

## Data Availability

The datasets generated and analysed during the current study are available in the Supplementary tables. All the sequencing data generated in this study have been deposited in NCBI SRA under accession number [BioProject ID: PRJNA1313160, submission: SUB15573960]. All data can be provided by the corresponding author after being requested.

## Abbreviations

HSD: High-salt diet
GF: Germ-free
NCDs: Chronic non-communicable diseases
CKD: Chronic kidney disease
ND: Natural diet group
BUN: Blood urea nitrogen
S-cr: Serum creatinine
TNFα: Tumor necrosis factor-α
IL-6: Interleukin-6
IL-1β: Interleukin-1β
IL-10: Interleukin-10
SIgA: Secretory Immunoglobulin A
NHE3: Na+/H+ exchanger type 3
SEM: Standard error of the mean
PCA: Principal-component analysis
PCs: Principal components
